# Supplementary material for: An Ice-Binding Protein from an Antarctic Ascomycete Is Fine-Tuned to Bind to Specific Water Molecules Located in the Ice Prism Planes
Source: Biomolecules. 2020 May 13;10(5):759. doi: 10.3390/biom10050759 (PMC7277481; doi:10.3390/biom10050759)
Supplement: Supplementary file 1 [file biomolecules-10-00759-s001.pdf]

biomolecules

MDPI

Supplementary Materials

An Ice-Binding Protein from an Antarctic Ascomycete Is Fine-Tuned to Bind to a Specific Set of Latticed Water Molecules Constructing an Ice Crystal Surface

Akari Yamauchi, Tatsuya Arai, Hidemasa Kondo, Yuji C. Sasaki and Sakae Tsuda

Table S1. Data collection and refinement statistics for wild-type *Anp*IBP and S153Y.

Crystal	AnpIBP (7BWX.pdb)	AnpIBP_S153Y (7BWY.pdb)
Data collection		
Beam line	Photon Factory BL-17A	
Wavelength (Å)	0.9800	
Space group	$P2_12_12$	$P3_221$
Unit-cell parameters (Å)	a = 86.43, b = 208.28, c = 100.78	a = b = 92.31, c = 22.67
Resolution range (Å) [a]	47.62–1.90 (2.01–1.90)	46.10–2.02 (2.06–2.02)
R_{merge} [a,b]	0.107 (0.467)	0.083 (1.654)
Observed reflections	944254	1442200
Independent reflections	141990	72989
Completeness (%) [a]	99.9 (99.6)	99.9 (99.8)
Multiplicity [a]	6.7 (6.6)	19.8 (19.6)
$\langle I/\sigma(I)\rangle$ [a]	12.9 (4.2)	21.1 (2.2)
Refinement		
Resolution range (Å) [a]	47.62–1.90 (1.95–1.90)	45.72–2.02 (2.07–2.02)
R factor [a,c]	0.135 (0.194)	0.179 (0.424)
Free R factor [a,c,d]	0.164 (0.216)	0.216 (0.418)
R.M.S bond lengths (Å)	0.013	0.011
R.M.S bond angles (°)	1.695	2.081

[a] Values in parentheses are for the highest resolution shell. [b] $R_{\mathrm{merge}} = \sum\sum_j \left|\langle I(h)\rangle - I(h)_j\right| / \sum\sum_j \langle I(h)\rangle$, where $\langle I(h)\rangle$ is the mean intensity of a set of equivalent reflections. [c] R factor = $\sum\left||F_{obs}(h)| - |F_{calc}(h)|\right| / \sum|F_{obs}(h)|$, where F_{obs} and F_{calc} are the observed and calculated structure factors, respectively. [d] Randomly chosen 5.0% of the reflection data were used to calculate free R factor (Brunger AT, 1992, Nature 355, 472–475 [39]).

A

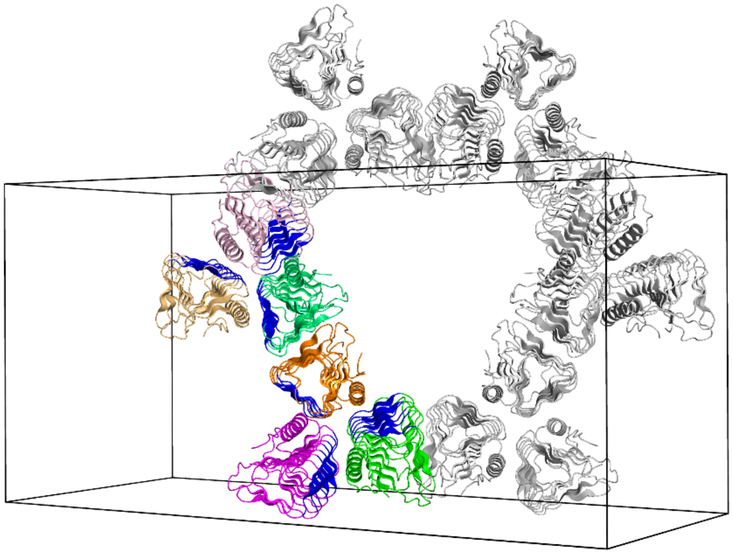

B

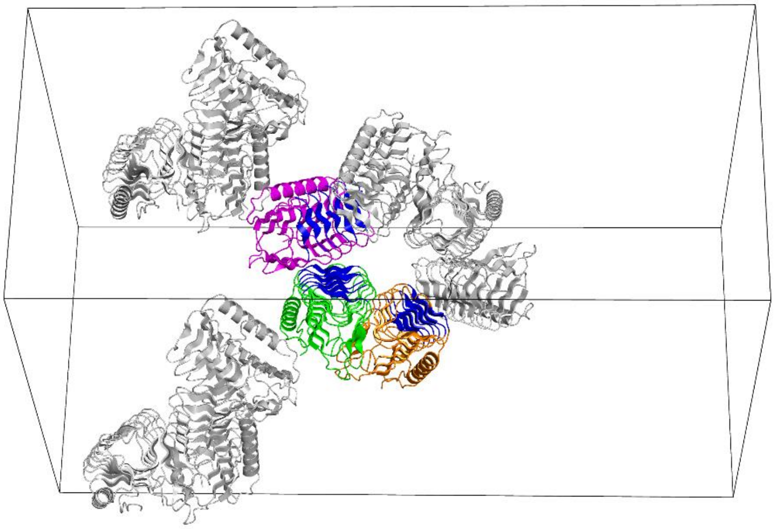

Figure S1. Crystal packings of wild-type *Anp*IBP and its mutant (S153Y). (**A**) Shows the molecular packing in wild-type *Anp*IBP crystal, which were crystallized in space group $P2_12_12$. Six monomers of *Anp*IBP in the asymmetric unit of the cell are drawn with color. IBS residues are colored blue. Neighboring molecules which are related by the crystallographic symmetry operations are drawn with gray. A unit cell is represented by black lines. (**B**) Shows the molecular packing for the S153Y mutant in the space group of $P3_221$. Three molecules in the asymmetric unit and neighboring molecules are drawn with the same color scheme as for the wild-type. For both wild-type *Anp*IBP and S153Y mutant, IBS residues are almost free from the molecular contacts and exposed toward the solvent. The figures were generated by Pymol.

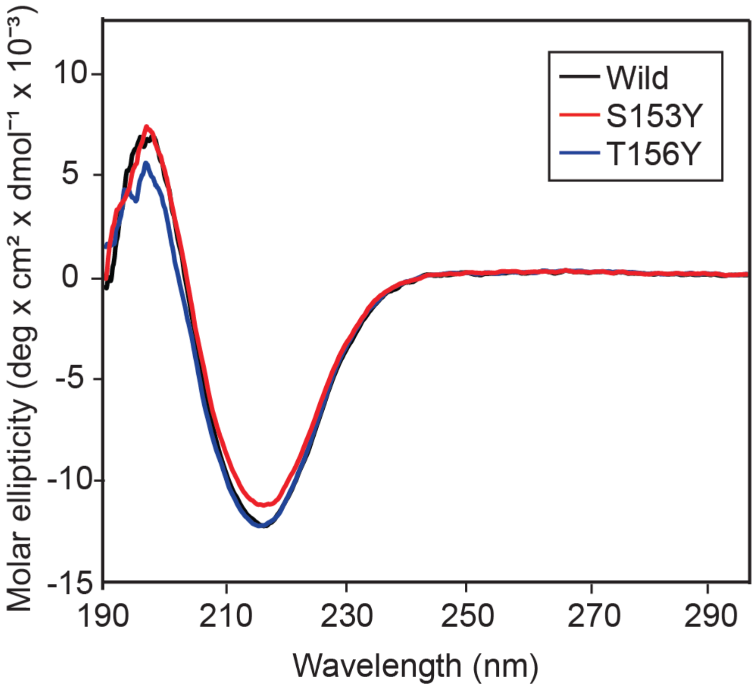

Figure S2. CD spectra of wild-type *Anp*IBP and its mutants (S153Y and T156Y). Circular dichroism (CD) spectra of *Anp*IBP wild-type and its mutants (S153Y and T156Y). The CD spectra were measured by with a J-720 spectropolarimeter (Jasco, Tokyo, Japan) with a quartz cuvette of 0.1 cm optical path length. *Anp*IBP and its mutants were dissolved in 10 mM sodium phosphate buffer (pH 7.4). Spectral data were collected from 190 and 260 nm at room temperature. We repeated the scans four times, and the averaged values were plotted.
